# Berlin Letter

**Published:** 1892-04

**Authors:** 


### Correspondence.

### BERLIN LETTER.

*Dr. Frank J. Thornbury discourses upon The Bacillus of Influenza.—The studies and conclusions of Dr. Pfeiffer at the Institute for Infectious Diseases.—The researches of Kitasato.*

DR. R. PFEIFFER, assistant to Professor Koch at the Institute for Infectious Diseases here, has made detailed and accurate bacteriological investigations in thirty-one cases of influenza, six of which afforded post mortem observation. The following are his results :

1. In all cases a certain form of bacillus was found in the characteristic purulent bronchial exudate. In pure cultures from all uncomplicated cases of influenza the presence of this bacillus was established, and in most instances myriads of the bacilli were present. Very frequently they were found situated within the protoplasma of the pus corpuscles. In patients attacked with influenza, who had previously been suffering from some affection of the respiratory apparatus, for instance, tuberculosis with excavation, other organisms are found in the expectoration in diffuse numbers. From the bronchi the bacilli may penetrate into the peri-bronchial connective tissue, and they may even succeed in reaching the visceral layer of the pleura, where they were found in pure cultures in two cases upon which autopsies were made.

2. These organisms are of *rod* shape, were found in cases of influenza *exclusively*. A vast number of control examinations established the fact that they are not present in ordinary cases of bronchitis, bronchial catarrh, pneumonia, and phthisis.

3. The number and presence of the bacilli are in direct relation to the course of the disease ; with the subsidence of the purulent bronchial secretion the bacilli also disappear.

4. The same bacilli in the same vast numbers occurring in the sputum of patients affected with influenza were observed and photographed by Pfeiffer two years ago when the disease first became epidemic.

5. The influenza organism is a rod-shaped *bacillus* of very diminutive size, having about the same thickness, but is only one-half as long as the mouse septicemial bacillus. Frequently three or four bacilli succeed one another in regular succession on the field, thus forming a sort of chain. With the basic aniline dyes there is considerable difficulty in staining the organism. A better preparation is found in a diluted Liehl's solution with Loffler's methyl-blue. Staining by this method it will be observed quite frequently that the end pole of the bacillus takes up the coloring matter to a much greater extent, so that an appearance is presented which may be easily confounded with diplo- or streptococci. It may, in fact, be accepted that one of the previous observers who also saw this bacillus described by Pfeiffer, by reason of this special peculiarity of staining, was deceived, and erroneously designated the germ diplo- and streptococci in his writing. The bacilli are not susceptible of staining by Gram's method. In hanging drop they have no motion.

6. They are permissible of maintenance in pure cultures. In $1\frac{1}{2}$ per cent. sugar-agar the colonies appear as exceedingly minute watery drops, often recognizable only by means of the "lupe." The further propagation of the germ in this medium is attended with difficulty, and beyond the second generation Pfeiffer has not been successful.

7. A number of attempts have been made to produce the disease in the lower animals. Apes, rabbits, guinea-pigs, rats, doves, and mice have been innoculated. These attempts have been successful only in case of apes and rabbits. The remaining species manifest a refractive tendency toward the disease.

8. In accordance with the foregoing facts, Pfeiffer maintains that the herein-described bacillus is to be accepted as the absolute cause of influenza.

9. The infection takes place in all probability through the bacteria-laden sputum, consequently as a prophylactic precaution the expectorate of all influenza patients must be thoroughly disinfected.

Kitasato has been successful in cultivating the influenza bacillus in sugar-agar to the fifteenth generation.

In presenting the subject before the Berlin Medical Faculty, at their last meeting, Kitasato preceded his remarks by stating: "It is surprising that such a long time should have elapsed before the specific infectious cause of influenza was found, considering the comparatively long time that the disease has prevailed, the vast endemic and epidemic proportions which it has assumed, and the numerous and widespread investigations which have been instituted."

The explanation according to Kitasato lies in the extreme difficulty in cultivating the bacillus. It is self-evident that without securing pure cultures a bacteriologist cannot arrive at definite conclusions with a newly-encountered pathogenic organism.

The difficulty in obtaining pure cultures from the sputum is dependent upon its multiple contamination with bacteria from the mouth and elsewhere.

By reason of the luxurious and rapid growth of these bacteria in our ordinary artificial culture mediums, the especially sought for germ is completely overgrown and covered up.

The greater the probability of this taking place is in proportion to the slowness of development in the colonies formed by the germ under consideration. This outgrowth by other organisms is well known in case of the tubercle bacillus.

In order to obviate this difficulty encountered in obtaining permanently pure cultures of tubercle bacilli from the sputum direct, Koch has not as yet made public any method by which he has, during these many years, been repeatedly successful. Notwithstanding repeated efforts, the same has been the experience of Kitasato in case of tubercle, and also as regards influenza. Kitasato has not as yet made known the method by which he has been successful in obtaining pure cultures of the influenza bacillus, but he promises very soon to do so.

In regard to the characteristics of the bacillus, his observations correspond exactly with those of Pfeiffer.

On glycerine-agar, in tubes coagulated obliquely, the individual colonies appear over the surface as exceedingly small, during the first twenty-four hours barely perceptible points resembling condensed vapor. So that macroscopically an inoculated tube is with difficulty distinguished from one sterilized.

As stated previously, the individual colonies are so exceedingly small and infinitely indistinct that they may be easily overlooked, and such, probably, has been the ill-luck of former investigators. One of these small colonies transferred to a fresh agar tube leads to the development of a multiplicity of colonies recognizable on the moist surface of the agar. Especially striking in the same is the fact that the individual colonies remain isolated and separate and distinct from each other, not aggregating and forming a connected covering over the surface, as do all other forms of bacteria in cultures. This isolated growth on agar is so characteristic that the influenza may be recognized with certainty from all other bacteria.

In gelatine the germ cannot be cultivated, as below 28° C. (the coagulation point of gelatine) they do not grow.

In bullion it grows scantily. In the first twenty-four hours, one recognizes, swimming in the bullion, white crumbly particles, the bullion itself remaining perfectly clear. Later, these small particles sink to the bottom and form there a white flocculent clump, the bullion above still remaining clear,—an evidence that the bacilli are devoid of motion. Kitasato has for a long time been examining tubercular sputum, and made accurate microscopic and culture study of all microörganisms found in association with the tubercle bacillus. The sputum of pneumonia and bronchitis cases has also been examined accurately and extensively, but at no time has this "so exceedingly characteristic and easily recognizable bacillus" been seen, excepting in cases of influenza.

P. Canon in examining the blood, per stained preparation, in twenty consecutive cases of influenza, found a particular form of microörganisms present in each instance.

The examinations were conducted in the following manner: One of the fingers of the patient is punctured with a needle in the usual manner, after the usual preliminary cleansing and aseptic precautions. The drop of blood which oozes is taken up in a very thin cover-glass, over which another is laid, and the two then suddenly are pulled apart. The cover-glasses are then allowed to dry, after which they are laid in absolute alcohol for five minutes. They are next placed in a coloring solution of the following composition (Czenynke fluid): concentrated aqueous methyline blue solution, 40 grains; one-half per cent. Eosin solution (in 70 per cent. alcohol dissolved), 20 grains; aq. dist., 40 gr.

In this solution the cover-glasses are placed in the culture chamber at 37° C., and here allowed to remain for three to six hours. They are then washed out in water, dried and mounted in Canada balsam.

In the blood preparation thus made the corpuscles are stained red, while the bacilli have a contrasting blue color.

Sometimes a great number of bacilli are present; again there are only a few, four to twenty, perhaps, in the entire field, and these are scattered and seen only after continued searching. At first they appear as diplococci; soon, however, (especially easy when deeply colored,) they will be recognized as short rods.

In six cases Canon found these bacilli in the preparation of blood in numerous large groups, containing from five to fifty bacilli each, and presenting a very characteristic appearance.

The blood was obtained in these six cases during the temperature elevation, or very soon after its decline. In three of the cases there was no further rise of temperature, and in six days there were no bacilli to be found.

In a patient of Professor Guttmann's, where the diagnosis was doubtful, Canon was able to state positively, by examination of the blood, that the case was one of influenza.

Also in other instances were the bacteria found in the blood, and even in great numbers where no local symptomatic evidence was present, especially no cough and expectoration.

Canon inoculated (per striate) tubes of agar, glycerine-agar, sugar-agar, and bullion, at the same time and from the same specimens of blood that he had examined microscopically.

In six cases the inoculated bullion was injected into mice, three at once, the remainder the following day, after which they were placed in a warm chamber. These injections, however, and examinations of the animals, gave negative results. [NOTE.—As previously stated, only monkeys and rabbits are susceptible to the disease according to Pfeiffer.]

In accordance with his investigations, Canon is of the opinion that this organism occurs in the blood in all cases of influenza (at least in all cases attended with fever), that it is not found in the blood of other cases, and that it stands, therefore, in direct causative relation to the disease.

Koch himself examined the blood preparations made by Canon, and pronounced the bacillus found in them identical with those found by his assistant Pfeiffer.

The blood investigations by Canon were begun about the middle of December, and he has still a great number of preparations to color and examine, and will thus continue his observations.

Last November, Lawrence demonstrated in Professor Nothnaggel's Clinic at Vienna, a microörganism found in the sputum of patients suffering with influenza.

The organism was a coccus, and resembled the Fraenkel-Weipselbaum diplococcus of pneumonia, excepting that it was much smaller. It had the Liehl stain, and appeared under the microscope as an ordinary streptococcus.

Sometimes a dozen or more of these organisms would be seen in the cells of the bronchial exudate, forming a quite perfect chain. They seemed to multiply with great rapidity, and were found in the blood; especially in cases of high temperature range were they numerous.

Lawrence regarded the germs as of an especially virulent disposition and pathogenic. He stated that we might with a reasonable degree of certainty accept them as the cause of the disease.

The reason of their appearing as diplococci is sufficiently explained by the peculiarity of staining, to which Pfeiffer directs attention. F. J. T.

63 Kloster Strasse, Berlin, January 28, 1892.

---

### NEW BUILDINGS FOR THE JEFFERSON MEDICAL COLLEGE OF PHILADELPHIA.

The Jefferson Medical College authorities have just completed the purchase of two large lots, on Broad street, upon which they will proceed to erect at once a handsome hospital, lecture hall, and laboratory building. The hospital will be built not only as a suitable building in which to care for the sick and injured, but also will be provided with a large amphitheatre for clinical lectures. By the erection of three commodious buildings, the laboratories, where delicate work with the microscope or apparatus is carried on, will be separated from the college hall, where didactic lectures are given, and so will be free from any jarring produced by the movement of large classes. With the hospital on one side, affording clinical facilities, and the laboratory on the other side of the college hall, for scientific research and training, the college will be most favorably situated for giving thorough instruction in medicine. The move has been made necessary by the large number of students who are now being instructed in this institution, and because the Faculty desire to keep the school and hospital in the foremost rank of medical education in this country. The building will be ready for occupancy in the session of 1893–'94.

